# Examining risk factors related to digital learning and social isolation: Youth visual acuity in COVID-19 pandemic

**DOI:** 10.7189/jogh.11.05020

**Published:** 2021-08-21

**Authors:** Ji Liu, Qiaoyi Chen, Jingxia Dang

**Affiliations:** 1 Faculty of Education, Shaanxi Normal University, Xian, Shaanxi, China; 2 School of Basic Medical Sciences, Xian Jiaotong University, Xian, Shaanxi, China; 3 The First Affiliated Hospital, Xian Jiaotong University, Xian, Shaanxi, China

## Abstract

**Background:**

Around the globe, various self-quarantine, social distancing, and school-closure policies were implemented during the coronavirus disease-19 (COVID-19) outbreak to reduce disease transmission. Many economies/territories were compelled to consider digital learning modalities. In particular, increased digital learning engagement with digital devices and mounting psychosocial stress due to social isolation are likely to pose adverse risks for youth visual health globally. This study examines the association between increased digital device use, psychosocial stress, and myopia symptoms among Chinese youth during the COVID-19 pandemic.

**Methods:**

This is a retrospective observational population study consisting of 3918 participants enrolled in primary, secondary, and university in China. Participants are recruited through an online survey, which included self-reported information on daily digital device use, psychosocial stress level, condition of visual acuity, and demographic information. We utilize statistical tools including χ^2^ test, paired sample *t* test, and multiple multivariate logistic regression.

**Results:**

Each hour increase in digital device use is associated with 1.25 odds ratio OR (95% confidence interval (CI) = 1.21-1.30; *P* < 0.001) increased risk of developing myopic symptoms, each additional hour of digital device use weighted by near-view and blue-light exposure is associated with OR = 1.04 OR (95% CI = 1.03-1.05; *P* < 0.001) and OR = 2.25 (95% CI = 1.94-2.60; *P* < 0.001) increased risk respectively. Subjects reporting under stressful conditions are between OR = 1.98 (95% CI = 1.67-2.36; *P* < 0.001) and OR = 2.03 (95% CI = 1.71-2.42; *P* < 0.001) more likely to develop myopic symptoms, relative to those citing less stress.

**Conclusions:**

The COVID-19 pandemic led to favorable conditions for myopigenic behavioral changes characterized by extended sedentary engagement with digital devices, which are significantly associated with higher risks of myopia incidence. Relatedly, psychosocial stress accompanying prolonged social isolation during the pandemic is a less noticeable, albeit significant risk factor for myopia development.

Myopia boom has become a global public health concern. While myopia is regarded a benign vision disorder that affects conditions of visual acuity, high myopia prevalence is shown to be growing at an alarming rate and its development has been found to be strongly associated with comorbidities that could result in irreversible vision loss, including cataract disorder, glaucoma, macular degeneration, posterior staphyloma, retinal detachment, and subretinal neovascularization [1]. Notably, approximately 1.406 billion (22.9% of global population) people around the world are myopic, and the number of affected is projected to increase to 4.758 billion (49.8%) by 2050 [2,3]. In the US and in Europe, about one third of adults are suffering from myopia [4,5], while in many East Asian countries, 80%-90% of young adults are estimated to be affected by myopic symptoms [6-8].

Risk factors for myopia development include age, gender, hereditary myopia condition, near vision work, electronic device use, sedentary time, and psychosocial stress [9]. Recent evidence has suggested that the coronavirus disease-19 (COVID-19) induced school-closures and home-confinement are positively correlated with progression of myopia in school-age adolescents [10]. Around the world, various self-quarantine and social distancing policies were implemented during the COVID-19 outbreak to reduce disease transmission. Despite the effectiveness in controlling the pandemic, these wide-ranging quarantine measures have fomented many unexpected consequences, including psychological stress due to social isolation and obesity as a result of limited outdoor activities. In addition, new evidence suggests that the pandemic has created favorable circumstances for promoting the development of refractive error among youth [11]. During the pandemic, school-age youth in more than sixty economies/territories faced widespread lockdowns and school-closures, and have to consequently resorted to remote digital learning arrangements [12]. On the one hand, strict social-distancing measures and home quarantine requirements promulgated by authorities strongly influence psychosocial stress, which can have consequential impact on youth’s mental and physical health [13]. On the other hand, remote digital learning arrangements have become common strategies to remediate adverse effects of pandemic-related school closures, but extended periods of digital device use and the associated exposure to near-view digital screens can be detrimental to young people’s visual development [14].

Critically, psychosocial stress has been identified as a key risk factor associated with psychosomatic reaction in the visual system [15], and symptomatic release of stress hormones can lead to vascular dysregulation [16] and influence vascular tone degradation around the optic nerve [17]. In pathological studies, acute psychosocial stressors were identified as key channels leading to increased vision loss risks, due in part to imbalanced autonomic nervous system functioning and spasm in ciliary muscles [18], and are associated with poor vision field performance [19]. Specifically, intensive eye use in a combination of stress hormone release [20], which can activate inflammatory responses and elevate the straining of tissues and muscles around the eye through neural activation of signaling pathways in immune cells [21]. In the context of significant disruption to regular school activities and elevated levels of social uncertainty during the COVID-19 pandemic, ocular impact as result of psychosocial stress among youth populations is likely more pronounced due to multiple overlap of school-closures and home-confinement. In this regard, the associated risks of vision irregularities as a result of widespread school disruptions during an ongoing pandemic demand greater health attention globally.

Additionally, the global transition to digital learning modalities during the COVID-19 pandemic is reasonably expected to compound the adverse visual health consequences of increased psychosocial stress among young people. For one, sharp increase in digital device engagement due to online instructional and learning requirements may expose young children to excessive near-view hyperopic defocus stimulation [22], which can trigger abnormal axial growth [23] and lead to premature refractive error development [24], particularly among youth whose visual system is experiencing rapid growth. For another, large and concentrated dose of blue light emitted from electronic screens can promote retinal pigment epithelium dysfunction [25], which can lead to destabilization of the outer blood-retinal barrier between neuroretina and the choroids [26] causing critical delays in retinal growth signals [27]. To this end, while existing physiological research suggests that both channels may contribute considerable myopic vision risks during a time characterized by new norms in learning arrangements, pre-COVID-19 pandemic population studies have suggested that use of electronic devices did not significantly increase risk of myopia among school-age youth [28].

In sum, although myopic visual acuity risks amidst increased digital device use and elevated psychosocial stress among youth during the COVID-19 pandemic has been a key global health concern, the magnitude of its adverse impact and the channels through which effects operate are not well understood. Leveraging a large-scale population survey, this study sets out to examine the potential vision implications as a result of increased digital device use and psychosocial stress among Chinese youth during the COVID-19 pandemic.

## METHODS

### Study design

This study was approved by the Shaanxi Normal University Institutional Review Board. Informed consent was obtained electronically from subjects, or parents/guardians of subjects. Between the months of January and May 2020, schools were closed nationwide in China due to the first wave of the COVID-19 pandemic. An online survey was conducted between May 12 to May 18, 2020 by *Teacher Daily* (*Jiaoshibao*), which is a nationally-known education press. Quality of the survey was ensured using Delphi approach, which included a panel of clinical ophthalmologist, education practitioner, public health researcher, who guided the design of instruments and sampling approach. Each questionnaire on average takes about 10-15 minutes to complete, and is intended to guide respondents to report their time use, visual acuity condition, and stress level information during the pandemic school-closures. Inclusion of subjects in this study were based on the following selection criteria: (1) could fully understand contents of the questionnaire; (2) presently enrolled in primary, secondary, or university; (3) voluntary participation; (4) submitted only one response using the same Internet Protocol (IP) address; (5) acknowledged informed consent. A total of 3918 subjects satisfied the inclusion criteria and are included in the analytic sample.

Information on sample descriptive statistics is presented in **Table 1**. Among 3918 subjects who are included in the analytic sample, 1536 (39.2%) reported symptomatic myopia development during the COVID-19 school closures. Prevalence of pre-pandemic myopia in the analytic sample is 57.8%. The analytic sample is comprised of 2072 (52.9%) female and 1845 (47.1%) male subjects, among which 2234 (57.0%) are enrolled in primary, 1171 (29.9%) are enrolled in secondary, 513 (13.1%) enrolled in university; 615 (15.7%) are located in rural areas, 392 (10.0%) are located in urban-rural transitional areas, and 2911 (74.3%) are located in urban cities. In terms of daily digital device use, subjects report an average of 3.91 unweighted hours (95% confidence interval (CI) = 3.83-3.98; standard deviation (SD) = 2.33). Once accounting for near-view eye use that vary by device type, the sample average rises to 14.29 near-view weighted hours (95% CI = 13.94-14.63; SD = 10.9), whereas the sample mean is 0.84 blue-light weighted hours (95% CI = 0.82-0.85; SD = 0.55). These numbers suggest that during the COVID-19 pandemic, digital device use duration is substantial among Chinese youth, especially accounting for near-view eye use interaction with devices such as smartphones. More importantly for psychosocial stress during the COVID-19 school-closure, 846 (21.6%) subjects reported as being “Stressful”, whereas 458 (11.7%) indicated that they consider themselves to be “Relaxed,” and the remaining 3027 (66.7%) self-report as indifferent between feeling stressed or relaxed.

**Table 1:** Sample descriptive information and univariate analysis results*

Variable	Total (Percent)	Incidence of myopic symptoms (Yes = 1, No = 0)		
**Count**	**Percent**	***P*-value**
Incidence of myopic symptoms:				
Yes	39.2	1536	-	-
No	60.8	2382	-	-
Sex:†				
Female	52.9	774	41.9	0.001
Male	47.1	762	36.8	
Level of study:†				
Primary	57.0	711	31.8	<0.001
Secondary	29.9	647	55.3	
University	13.1	178	34.7	
Location of residence:†				
Rural	15.7	238	38.7	<0.001
Urban-rural	10.0	113	28.8	
Urban	74.3	1185	40.7	
Pre-pandemic myopia condition:†				
Yes	57.8	943	57.1	<0.001
No	42.2	593	26.2	
Daily digital device use, unweighted hours (mean, s.d.)‡	3.91, 2.33	mean (1)–mean (0) = 1.54		<0.001
Daily Digital Device Use, near-view weighted hours (mean, SD)‡	14.29, 10.9	mean (1)–mean (0) = = 5.99		<0.001
Daily Digital Device Use, blue-light weighted hours (mean, SD)‡	0.84, 0.55	mean (1)–mean (0) = 0.34		<0.001
Psychosocial stress:†				
Stressful	21.6	510	60.4	<0.001
Relaxed	11.7	100	21.8	
Indifferent	66.7	926	35.4	

SD – standard deviation

*In the first analytic stage, we report a series of bivariate associations between subjects’ background information and vision condition during the COVID-19 school-closures using univariate analysis.

†*P*-value based on χ^2^ test.

‡*P*-value based on *t* test.

### Survey and measures

The survey questionnaire guided subjects through a series of questions to self-evaluate changes in their vision condition using the Lay Terms Approach, anchoring on an inventory of subjects’ familiar vocabulary [29], including questions such as whether “squinting was needed to see clearly.” The survey collected information on time (hours) spent using digital learning devices (TVs, computers, or smartphones), as well as psychosocial stress ratings (stressful, relaxed, or indifferent). In addition to reporting raw hours of daily digital device use, we also provide information on near-view weighted eye use (diopter hours, dh) and blue-light radiance weighted exposure (h • W m^−2^ sr ^−1^). Following prior research that had weighted near-view eye use [30], near-view digital device use is categorized into three device groups: near (0.2 m for smartphone), intermediate (0.5 m for computer), and far (1.0 m for TV), and assigned inverse weights of 5, 2, 1 respectively shown in Equation 1. At the same time, we leverage experimental evidence on blue-light emission from different digital devices [31], and compute radiance weighted exposure hour by device type, in the following sequence: TV (0.08 W m^−2^ sr ^−1^), computers (0.16 W m^−2^ sr ^−1^), smartphones (0.26 W m^−2^ sr ^−1^), which is also shown in Equation 2 as a weighted function of mean blue-light radiance for specific device type and hours of use.

Equation 1: Near-view weighted eye use = (5 × hours viewing at 0.2 m) + (2 × hours viewing at 0.5 m) + (1 × hours viewing at 1.0 m)

Equation 2: Blue-light weighted eye use = (0.26 W m^−2^ sr^−1^ × hours viewing at 0.2 m) + (0.16 W m^−2^ sr^−1^ × hours viewing at 0.5 m) + (0.08 W m^−2^ sr^−1^ × hours viewing at 1.0 m)

### Statistical analysis

There are two inter-related analytic stages in this study. In the first analytic stage, univariate analyses are conducted, and we report χ^2^ test and paired sample *t* test results. Importantly in this stage, we assess to what extent self-reported myopic vision condition (dependent variable) varies by individual characteristics. A *P* value of <0.05 was considered to be statistically significant. In the second analytic stage, multivariate logistic regression models are employed to examine the association between digital device use, psychosocial stress, and myopia development, upon controlling for personal traits and pre-pandemic vision condition. We draw upon prior studies to determine the list of control covariates [9], which include information on subjects’ sex, level of study, location of residence, and pre-existing myopic condition. Level of study is categorized into three groups: primary, secondary, or university, while location of residence is defined as rural, urban-rural (transitional areas), or urban. All analyses were performed using STATA version 15.0 (Stata, StataCorp LLC, College Station, TX) software.

## RESULTS

### Predictors of myopia development

First, findings show that girls are on average more likely than boys to report loss in visual acuity during the COVID-19 pandemic school-closures (χ^2^ = 10.87, *P* = 0.001). Second, subjects enrolled in secondary schools are more likely to report vision deterioration issues than those enrolled in either primary or university (χ^2^ = 185.68, *P* < 0.001). Third, subjects residing in urban-rural transitional areas report markedly lower rates of myopic symptoms than those in either rural or urban areas (χ^2^ = 20.54, *P* < 0.001). Fourth, subjects who already suffer from myopia prior to the COVID-19 pandemic appear to experience higher rates of symptomatic myopia development during the school closures (χ^2^ = 381.99, *P* < 0.001). Fifth, the average duration of daily digital device use is significantly higher for subjects who experienced vision issues during the pandemic, at 1.54 hours (*P* < 0.001), 5.99 diopter hours (*P* < 0.001), 0.34 hours × W m^−2^ sr^−1^ (*P* < 0.001) for unweighted, near-view weighted, and blue-light weighted hours respectively. Sixth, subjects who experience psychosocial stress also indicate higher incidence of myopic symptoms than those who report either relaxed or indifferent (χ^2^ = 232.37, *P* < 0.001).

### The association between digital device use, psychosocial stress, and myopia development

In **Table 2**, we divide the analysis into three different models, each with a different measure of digital device use duration: unweighted (Model 1), near-view weighted (Model 2), blue-light weighted (Model 3). This differentiation enables us to understand through which channels the adverse vision risks of digital device use is directed, namely digital device engagement, near-view hyperopic defocus, or electronic blue-light emission. Importantly, findings across all three models indicate that daily digital device use is positively associated with myopic risks, regardless of using weighted or unweighted exposure measures. More specifically, every one-hour increase in unweighted digital device use is associated with odds ratio (OR) = 1.25 (95% CI = 1.21-1.30; *P* < 0.001) increased incidence of myopic symptoms, while each near-view and blue-light weighted hour is associated with OR = 1.04 (95% CI = 1.03-1.05; *P* < 0.001) and OR = 2.25 (95% CI = 1.94-2.60; *P* < 0.001) increased risk respectively. By comparing the OR coefficients for the typical subject in the sample, who on average report 3.91 unweighted hours, 14.29 near-view weighted hours, and 0.84 blue-light weighted hours, it can be observed that the associated risk is largest for near-view weighted measure of digital device exposure (OR = 14.86), followed by unweighted (OR = 4.89) and blue-light weighted (OR = 1.89) respectively.

**Table 2:** Multivariate logistical regression analysis and model comparison results*

Variables	DV: Incidence of myopic symptoms (Yes = 1, No = 0)								
**Model 1 (Unweighted hours)**			**Model 2 (Near-view weighted hours)**			**Model 3 (Blue-light weighted hours)**		
**OR**	**95% CI**	***P*-value**	**OR**	**95% CI**	**P**	**OR**	**95% CI**	***P*-value**
Daily digital device use	1.25	1.21-1.30	<0.001	1.04	1.03-1.05	<0.001	2.25	1.94-2.60	<0.001
Psychosocial stress level:									
Stressful	1.98	1.67-2.36	<0.001	2.03	1.71-2.42	<0.001	2.00	1.68-2.37	<0.001
Relaxed	0.64	0.50-0.82	<0.001	0.64	0.50-0.82	<0.001	0.65	0.51-0.83	0.001
Indifferent	1			1			1		
Sex:									
Female	1.02	0.89-1.18	0.746	1.05	0.91-1.21	0.507	1.03	0.90-1.19	0.662
Male	1			1			1		
Level of study:									
Primary	1.76	1.37-2.24	<0.001	1.42	1.12-1.79	0.003	1.55	1.23-1.97	<0.001
Secondary	1.33	1.04-1.70	0.022	1.42	1.11-1.80	0.005	1.36	1.07-1.73	0.013
University	1			1			1		
Location of residence:									
Rural	1.06	0.87-1.29	0.564	1.06	0.87-1.29	0.564	1.04	0.86-1.27	0.679
Urban-rural	0.78	0.60-0.99	0.044	0.75	0.58-0.97	0.029	0.76	0.59-0.99	0.039
Urban	1			1			1		
Pre-pandemic myopia condition									
Yes	2.60	2.22-3.05	<0.001	2.75	2.34-3.23	<0.001	2.66	2.27-3.13	<0.001
No	1			1			1		

OR – odds ratio, CI – confidence interval, DV – distance vision

*In the second analytic stage, we investigate the association between digital device use, psychosocial stress, and myopia development, after controlling for personal traits and pre-pandemic vision condition leveraging multivariate logistic regression.

In addition, higher levels of psychosocial stress is shown to be positively associated with risks of myopia development, such that “Stressful” subjects are between OR = 1.98 (95% CI = 1.67-2.36; *P* < 0.001) and OR = 2.03 (95% CI = 1.71-2.42; *P* < 0.001) more likely to experience myopic symptoms, than those who report indifference. On the contrary, subjects who report being “Relaxed” are between OR = 0.64 (95% CI = 0.50-0.82; *P* < 0.001) and OR = 0.6 (95% CI = 0.51-0.83; *P* < 0.001) less likely to develop myopic symptoms, relative to those who report indifference.

Last but not least, results for subject background information also indicate several generalizable patterns. First, girls are no more likely than boys to experience myopic symptoms across all model specifications (*P* = 0.746, *P* = 0.507, *P* = 0.662). Second, subjects enrolled in primary are between OR = 1.76 (95% CI:1.37-2.24; *P* < 0.001) and OR = 1.42 (95% CI = 1.12-1.79; *P* = 0.003) more likely than subjects in university to exhibit myopic symptoms; the same is true for subjects enrolled in secondary with slightly smaller odds ratios. Third, there is no discernable difference in myopic symptoms between rural and urban subjects across all model specifications (*P* = 0.564, *P* = 0.564, *P* = 0.679), but subjects in urban-rural transitional areas are about OR = 0.75 (95% CI = 0.58-0.97; *P* = 0.029) and OR = 0.78 (95% CI = 0.60-0.99; *P* = 0.044) less at risk than those in urban areas. Fourth, pre-pandemic myopia condition among subjects is positively associated with myopia development, for which it is estimated to vary between OR = 2.60 (95% CI = 2.22-3.05; *P* < 0.001) and OR = 2.75 (95% CI = 2.34-3.23; *P* < 0.001).

## DISCUSSION

Poor vision health can have extensive impacts on society as its associated consequences are often correlated with reduced quality of life and elevated digital health concerns. In this regard, the digital health risks stemming from favorable environmental factors affecting visual acuity have been less documented [32], particularly during the COVID-19 pandemic when such factors have become increasingly salient. In this study, findings show that duration of digital device use is reported to be on average 3.91 hours per day in our sample, which significantly exceeds the World Health Organization digital screen use duration recommendation limit of 2 hours/d for children aged between 5 to 17 years old [33]. Consequentially, each additional hour of digital device use is positively associated with 1.25 OR (95% CI = 1.21-1.30; *P* < 0.001) increased risk of myopia symptoms. Results are categorically similar after weighting digital device use by near-view distance and blue-light emission, indicating that more intensive sedentary use of electronic devices is linked to elevated risks of myopia development. In addition, the relative risks appear to be higher if weighting digital device use by near-view exposure, as compared to unweighted and blue-light weighted measures. These findings suggest that close proximity use of electronic devices may be a main channel through which myopigenic behavioral changes can elevate youth visual health risks. In broad strokes, this is consistent with prior studies that examine near-view health risks of digital devices [34], although several prior studies have highlighted that extended blue-light exposure presents key adverse clinical implications [35]. In this regard, youth and parental awareness of the risks of near-view digital device use should be broadly communicated, and public health recommendations on good eye use habits such as frequent eye breaks and timed device use intervals should be made widely available to youth and their families. This would be particularly meaningful considering that COVID-19 pandemic-induced remote learning is still on-going in many territories/economies. To this end, this study contributes large-scale survey evidence on the association between digital device use, psychosocial stress, and myopia development.

Despite global concerns and practitioner warnings against the adverse prospects of a looming myopia epidemic amidst the COVID-19 pandemic [1], there exists few population-based studies that explore the magnitude of emerging myopigenic risks among youth as result of heightened psychosocial stress due to social isolation. Findings in this study uncover the critical psychosocial health consequences of the pandemic on visual health, by showing that psychologically stressful condition is associated with approximately OR 1.98 (95% CI = 1.67-2.36; *P* < 0.001) increased risk of developing myopic symptoms. Prior studies have suggested that acute psychological stress may have a role in driving environmentally derived myopia incidence, corroborating our findings in this study. Complementing prior research that have yet to engage in jointly assessing psychosocial and physio-behavioral pathways [36], findings in this study imply that the less noticeable psychosocial consequences of the pandemic on youth visual health are on par, if not more influential, than its physio-behavioral counterparts. In this regard, it is critical to realize that school closures and home confinement can prompt psychosocial stressors, and home remedying interventions through frequent verbal interactions may be necessary to avoid developing posttraumatic complication [37].

Finally, a limitation in this study worth pointing out is relying on subjects’ self-evaluation of vision status, rather than conducting specialist eye examination, which would be ideal to detect refractive error. Since the initial COVID-19 outbreak, ophthalmologist availability has reduced significantly [38], and clinic visits have been particularly less viable. Under logistical circumstances and social-distancing requirements propagated by the COVID-19 pandemic, this study elects a rapid survey approach, which has been suggested by prior studies to be reasonably reliable for large samples [39]. In addition, the election to adopt a rapid online survey design may also have introduced nonrandom sampling bias, since the voluntary nature of survey enrollment creates a selection challenge common to many observational studies, particularly favoring those who live in urban areas, have access to internet and digital devices. Relatedly, further studies in the future may consider leveraging representative household- and phone-based eye examination survey approaches that are developed since the initial COVID-19 outbreak [40], in complementing data solicited through subjects’ self-evaluation.

## CONCLUSION

The scale at which the COVID-19 pandemic has affected livelihoods globally is unprecedented, particularly for an entire generation of youths coping with drastic changes in their daily lives. The less noticeable vision health risks of the pandemic are likely significant considering that school closures are widespread and on-going. These implications are especially relevant for youths, whose eyes are continuing to develop. Critically, alternative digital learning arrangements at home and myopigenic behavioral changes that are characterized by extended sedentary engagement with digital learning devices at close proximity will inevitably create critical risk factors for vision deterioration among school-age youth. In addition, psychosocial stress as a result of extended home confinement and social isolation is another crucial risk factor driving decline in visual acuity among youth. While our study provides an initial assessment of the potential risk factors of digital device use and psychosocial stress on myopia during the global COVID pandemic, further studies are needed to confirm the generalizability of these findings. Last but not least, future school-closure and delay-opening policies should be cognizant of the health impacts of extended dependence on digital learning modalities, and design innovative activity-based mitigation and low-tech alternative learning strategies [41], in order to minimize the longer-term collateral vision consequences on an entire generation of youth that is affected.
